# The Relationship Between Nutritional Anemia and Acne: A Case-Control Study

**DOI:** 10.7759/cureus.39109

**Published:** 2023-05-16

**Authors:** Norah M Alharbi, Fatimah A AlGhofaili, Jolan S Alsaud, Lina M Asiri, Shumukh M Almutairi, Dalal M Alruqayi, Maha K Alharbi

**Affiliations:** 1 College of Medicine, Qassim University, Qassim, SAU; 2 Dermatology, Qassim University, Buraydah, SAU

**Keywords:** saudi arabia, qassim region, relationship, nutritional anemia, acne vulgaris

## Abstract

Background

In the past decade, there has been growing interest in identifying the relationship between nutritional status and acne. Many dietary factors have been studied, including milk, fast food, and chocolate. However, nutritional anemia, which is a common problem in young individuals, has not been well investigated.

Objectives

The objective of this study was to determine the relationships between acne and nutritional anemia among people in the Qassim Region of Saudi Arabia.

Methods

This study used a case-control design. It targeted people aged from 15 to 25 years old in the Qassim Region of Saudi Arabia. This study was conducted using a database of Electronic Health Records (EHR) data from the Qassim University outpatient department (OPD). Data analysis was conducted by using SPSS (IBM Inc., Armonk, New York).

Results

A total of 114 of the study population were involved in this study. The acne group represented was identical to the control group. The mean age of study participants was 23.1 ± 4.19 years old, and the majority of them were females (86%). Moreover, the patient group had relatively lower levels of mean corpuscular volume (MCV), vitamin B12, ferritin, mean corpuscular hemoglobin (MCH), and hemoglobin than the control group without any significant correlation, whereas the patient group had higher levels of mean corpuscular hemoglobin concentration (MCHC) and red cell distribution width (RDW) without significant difference. Our results showed that the prevalence of anemia among respondents was 17.5%, and a higher prevalence of anemia was observed in the control group without significant differences. Furthermore, the patient group had a significantly higher prevalence of vitamin B12 deficiency (38.6%) than the control group (p=0.041).

Conclusion

Our results concluded that patients with acne vulgaris in the Qassim Region of Saudi Arabia had a significantly higher rate of vitamin B12 deficiency. Additional studies are needed to confirm this association.

## Introduction

Acne is a common skin ailment that affects approximately 9.4% of the world's population, with teens being the most affected. Across all ethnic groups, it affects more than 80% of females and 90% of males [1-2]. Although acne is neither physically debilitating nor life-threatening, it can have an influence on one's quality of life (QOL). Post-inflammatory discoloration and/or fibrotic scarring from moderate to severe acne lesions can lead to low self-esteem and social isolation [3]. It can also create depression, worry, and other types of emotional distress, all of which can have a significant influence on one's quality of life [4]. Nutritional anemia is a major public health issue worldwide, caused by a lack of essential nutrients such as iron, folate, and vitamin B12 [5-6].

The connection between nutrition and acne has been studied for many years. Acne appears to develop more quickly in people who consume a diet with a higher glycemic index [7-8]. Various vitamin supplements, most notably vitamin B12 (cyanocobalamin), have also been documented to aggravate existing acne and/or trigger the formation of acneiform outbreaks [9]. However, new research has shown that oral isotretinoin (ISO) treatment, which is effective against the key etiological variables implicated in acne development, may cause a drop in vitamin B12 and folic acid levels [10-11]. Over the last decade, there has been an increasing interest in determining the correlations between nutritional conditions and acne. Many dietary components, including milk, fast food, and chocolate, have been explored [12]. According to a study of contemporary scientific research, diets can influence skin problems in a variety of ways. However, in many circumstances, evidence-based nutritional recommendations are still unavailable [13].

Acne vulgaris is a persistent inflammatory condition that affects the pilosebaceous units. Most people will experience this syndrome at some point throughout their lives, particularly around adolescence [14]. Acne has been linked to a variety of environmental and internal variables, including air pollution, aggressive skin care products, medicine, mechanical, hormonal, and family factors, and, more recently, lifestyle and stress [15]. Increased levels of emotional stress can play a major etiological role in adult acne, leading to an increase in adrenal androgens [16]. There has been little research into the relationship between acne vulgaris and nutritional anemia.

A previous study in Turkey found no link between post-adolescent acne and nutritional anemia, contrary to a recent study in Turkey. Serum folate levels, on the other hand, were reduced in post-adolescent acne sufferers [17]. Furthermore, another study in Malaysia found that the relationships between acne vulgaris and chocolate and milk consumption support the concept that dietary factors may influence the development of acne vulgaris [18].

Another study that demonstrated the possibility of anemia developing after acne treatment detailed a case of anemia caused by a B12 and folate deficiency that manifested eight weeks after receiving isotretinoin treatment for acne. [19]. Leyden discovered that isotretinoin treatment for severe cystic acne was effective because it corrected anemia and low serum iron levels [20]. According to a previous study, some nutritional variables, a compromised antioxidant defense system, and changed intestinal microflora may interact to raise the risk of psychological sequelae in acne vulgaris patients with regard to their psychological status [21].

Acne has also been connected to a number of nutritional supplements. Numerous case studies and series have discussed how some dietary supplements might cause acne to appear, which then goes away when the supplements are stopped. Supplements can share chemicals with the aforementioned acne-causing medications, such as kelp and vitamin supplements that contain iodine [15,22]. Another study conducted in the United States revealed that a number of nutritional supplements, including those containing the vitamins B6, iodine, whey protein, and "muscle-building supplements," have been linked to acne [23].

The link between nutrition and acne was eventually disproved in 1969 when clinical research discovered that eating chocolate did not worsen acne lesions when compared to eating a placebo bar [24]. This study has drawn criticism for a number of design issues, including the comparable nutrient makeup of the chocolate bar and the placebo, despite the fact that it is the most frequently referenced source of dissociating diet and acne [25].

For the management of acne as well as to inform the public about prevention and therapy, a thorough knowledge and comprehension of the relationship between dietary factors and acne vulgaris are essential [26]. These will help dermatologists address any myths and misconceptions and offer patients well-supported dietary advice [27]. 

In 2013, a Jeddah-based survey aimed to explore the public's knowledge and views on acne in Saudi Arabia. The findings showed that 64.5% of respondents had acne, with 28.4% believing that diet was the most crucial factor in its development. In a more recent study in Riyadh, 78% of participants aged 15-30 experienced acne, with a higher prevalence in women (86.1%) than men (69.9%; p=0.001). Additionally, among medical students in Saudi Arabia, acne prevalence was observed to be 55%, with a majority (87.2%) attributing the condition to hormonal imbalances [28].

Only a few studies have investigated the link between nutritional anemia and acne. One of them is a case-control study conducted in 2013 in the United States among 52 reproductive-age women with acne and 52 healthy control population that found no significant connections between post-adolescent acne severity and nutritional anemia [17]. Another study, conducted in Philadelphia in 1985 on twenty-four males with severe nodulocystic acne who were referred for isotretinoin medication, revealed that nutritional anemia is related to the clinical state of cutaneous inflammation [20].

However, nutritional anemia, a prevalent condition in young people, has received little attention. We believe nutritional anemia has a role in the etiology of acne. In our study, we wanted to look into the relationship between acne and nutritional anemia.

## Materials and methods

Study Design

A case-control design was used to identify the association between acne and nutritional anemia. This study was carried out in the Qassim Region of Saudi Arabia. This study was conducted in 2022-2023, and it targeted people aged from 15 to 25 years old in the Qassim Region of Saudi Arabia.

Definition of cases and controls

In this study, cases are people aged from 15 to 25 years old living in Qassim who have been diagnosed with acne vulgaris at the dermatology department of Qassim University outpatient department (OPD) from January, 2022 to August, 2023. The controls were individuals aged 15 to 25 living in Qassim who had attended a dermatology clinic and had never been diagnosed with acne. They were matched based on age and gender.

Inclusion and exclusion criteria

People aged from 15 to 25 years old who live in Qassim Region were included in the study. The presence of chronic disease (e.g., hypertension, diabetes), and the presence of hematological problems and drugs Penicillin and its derivatives, cephalosporins, dapsone, Quinidine, etc., that may cause anemia in both acne and control groups were the exclusion criteria for this study.

Methods for data collection

This study was conducted using a database of Electronic Health Records (EHR) data from Qassim University OPD, with approval from the Qassim University Medical Institutional Review Board. EHR data used consisted of structured medication data, coded demographic information, laboratory results, and diagnosis codes, all from the period January 1, 2022, to August 30, 2023.

The list of variables included age, gender, acne, hemoglobin (g/dL), mean corpuscular volume (MCV; fL), mean corpuscular hemoglobin (MCH; pg), mean corpuscular hemoglobin concentration (MCHC; g/dL), red cell distribution width (RDW; %), ferritin (ug/L), and vitamin B12 (pmol/L)

Data management

Data was identified initially and then coded in the database Excel sheet (Microsoft, Redmond, Washington) using a unique identification number. Only the research team had access to the database for analyses purpose. The publication presented summary statistics, and no identifying information was used. The name that was used for determining the current health status was kept in a separate database, so it cannot be linked with any medical data. Values below 12 g/dL of serum hemoglobin for females and below 13g/dL for males were defined as anemia. Values below 11 ug/L of ferritin were defined as ferritin deficiency. Values below 107 pmol/L of serum vitamin B12 were defined as vitamin B12 deficiency.

Data analysis

Data was collected into the Statistical Package for Social Sciences (SPSS, version 23.0; IMB Inc., Armonk, New York). All numerical variables were expressed as mean and SD, while categorical variables were expressed as frequency and percentage. Independent samples t-test, chi-square test, and Mann-Whitney test were used in our study. A p=value of <0.05 was accepted as significant.

Ethical consideration

Regional review board approval and Qassim University Research Ethics Committee was obtained for this study before carrying on with it (21-22-07). All the information was treated in a confidential manner. The names and ID numbers of participants were kept in a special form, and destroyed after data collection was completed.

## Results

Characteristics of the study participants

In this study, we included 114 respondents. The patient group made up fifty percent of the study population as well as the control group. Overall, the mean age of study participants was 23.1 ± 4.19. Moreover, our results found that female participants represented the majority of the study population (86%), whereas males represented only 14%. The patient and control groups were identical according to age and gender (Table 1).

**Table 1:** Characteristics of the study participants

Variable	Overall (n=114)	Patient group (n=57)	Control group (n=57)	p-value
Age, mean ± SD (range)	c (15-36)	23.1 ± 4.21	23.1 ± 4.21	1
Gender, n (%)				
Male	16 (14%)	8 (14%)	8 (14%)	1
Female	98 (86%)	49 (86%)	49 (86%)	

Laboratory findings of the acne vulgaris group and control group

Our study conducted many laboratory investigations to measure the parameters of anemia in both patients and the control group. There was no significant difference between the patient and control groups regarding the level of anemia parameters. Our findings demonstrated that the patient group had a lower level of MCV than the control group (82.76 ± 5.70 and 84.12 ± 4.56, respectively) without any significant difference (p-value=0.318). Moreover, the patient group had relatively lower levels of vitamin B12, Ferritin, MCH, and hemoglobin than the control group without any significant correlation (p-values= 0.369, 0.542, 0.755, and 0.954). On the other side, patient group had higher levels of MCHC and RDW without significant difference (p-values= 0.358 and 0.762), as presented in Table 2.

**Table 2:** Laboratory findings of the acne vulgaris group and control group MCV- mean corpuscular volume; MCH - mean corpuscular hemoglobin; MCHC - mean corpuscular hemoglobin concentration; RDW - red cell distribution width

Characteristic	Overall (n=114)	Patient group	Control group	p-value
Hemoglobin (g/dl)	13.20 ± 1.46	13.20 ± 1.52	13.21 ± 1.40	0.954
MCV (fL)	83.44 ± 5.18	82.76 ± 5.70	84.12 ± 4.56	0.318
MCH (pg)	28.29 ± 2.24	28.14 ± 2.48	28.45 ± 1.99	0.755
MCHC (g/dl)	33.88 ± 0.92	33.96 ± 0.97	33.80 ± 0.88	0.358
RDW (%)	13.83 ± 1.43	13.88 ± 1.52	13.76 ± 1.34	0.762
Vitamin B12 (pmol/L)	148.70 ± 73.97	144.67 ± 73.47	152.74 ± 74.91	0.369
Ferritin (ug/L)	26.66 ± 28.18	27.42 ± 31.36	25.90 ± 24.84	0.542

Comparison of ferritin, vitamin B12 deficiency, and anemia between both groups

Additionally, our results showed that the prevalence of anemia among respondents was 17.5%. However, there was no significant difference between the acne and control groups (p-value=0.622). The patient group had a significantly higher prevalence of vitamin B12 deficiency (38.6%) than the control group (p-value=0.041). About one-third of participants had ferritin deficiency (33.3%), and it was particularly higher among the patient group (35.1%) than the control group (31.6%) without any significant association (p-value=0.691) (Table 3).

**Table 3:** Comparison of Ferritin, vitamin B12 deficiency, and anemia between both groups

Characteristic	Overall (n=114)	Patient group (n=57)	Control group (n=57)	p-value
Anemia	20 (17.5%)	9 (15.8%)	11 (19.3%)	0.622
Vitamin B12 deficiency	34 (29.8%)	22 (38.6%)	12 (21.1%)	0.041
Ferritin deficiency	38 (33.3%)	20 (35.1%)	18 (31.6%)	0.691

## Discussion

The current study aimed to assess the relationship between acne vulgaris and nutritional anemia among people from the Qassim Region of Saudi Arabia. Although teenagers frequently have acne, the overall rates seem to be rising, especially among adults [29-30]. Hyperseborrhea and hyperkeratinization, which appear to be common in this form of acne, can be caused by a number of environmental pathogenetic variables, including stress, atmospheric pollution, factors connected to one's employment and the environment, and photoexposure [31]. 

Our results showed that the prevalence of anemia among respondents was 17.5%. A relatively higher prevalence of anemia was observed in the control group without significant differences. Another study in Iraq showed almost similar results, which stated that serum iron level in the patient group was lower than in the control, but it was not statistically significant [32]. Furthermore, a second study from Turkey supports our findings, stating that there were no significant variations in hemoglobin levels between the two groups [17]. A previous study found that 30 people with moderate acne vulgaris, grade II, had serum levels of copper, iron, and zinc. The outcomes were contrasted with those from a control group that was healthy. Although they were statistically insignificant, the data showed variations in the sera's copper and iron concentration [33]. This suggested that anemia due to iron deficiency is not significantly related to acne vulgaris. 

Our findings demonstrated that the acne group had a significantly higher prevalence of vitamin B12 deficiency (38.6%) than the control group (p-value=0.041). This was inconsistent with another survey, which showed that serum vitamin B12 levels were increased in post-adolescent acne patients, but the difference was not statistically significant. In addition, vitamin B12 deficiency was significantly higher in the control group, while it revealed that serum folate levels were significantly decreased in post-adolescent acne patients (p<0.001) [17]. On the other hand, a different study found that B12 insufficiency emerged following the treatment of acne vulgaris. They described a case of anemia brought on by B12 and folate deficiencies that emerged eight weeks following Accutane treatment for acne [19]. Contrary to our findings, a different investigation reported that the patient group's pre-treatment vitamin B12 readings were statistically considerably higher [34]. An earlier study conducted in the USA revealed that supplementing with high dosages of vitamins B6 and B12 has been linked to complaints of acne worsening more frequently in females than in males [35]. However, it is important to mention that our study did not include the dietary supplementation of participants. These results support the assertion that vitamin B12 plays a role in the pathogenesis of acne vulgaris. Furthermore, About one-third of participants had ferritin deficiency (33.3%), 35.1% in the acne group and 31.6% in the control group, without any significant association. These findings are in accordance with another study that reported that there was a non-significant difference between patients' and control patients' serum ferritin levels [36]. It may be possible to identify numerous indicators that point to probable nutritional acne-causing variables. Therefore, if they can be identified, some individuals may be able to have their acne better managed, adding dietary control as another tool in the acne treatment toolbox.

Our study has some limitations. First, the study population was rather small, which could limit the generalizability of our findings. Second, it was single-centered, and medications were not accounted for. The latter is important because earlier, it was stated that isotretinoin is linked to vitamin B1 deficiency. The third missing limitation is that the study did not mention the severity of acne in the acne group. Most of the included patients had mild acne; this might explain the negative results of the study. Furthermore, we did not look into whether or not individuals were taking dietary supplements.

## Conclusions

Our results concluded that patients with acne vulgaris in the Qassim Region of Saudi Arabia had a significant relationship with vitamin B12 deficiency. This data suggested that nutritional anemia may play a role in the etiology and pathogenesis of acne vulgaris.
